# Chronic Edema Associated with Cor Pulmonale in Cattle

**DOI:** 10.3390/ani15172501

**Published:** 2025-08-25

**Authors:** Laís G. Caymmi, Múcio F. F. Mendonça, Paula V. Leal, Luciano A. Pimentel, Jose C. de Oliveira-Filho, Tiago C. Peixoto, Ana C. S. N. Souza, Ricardo B. de Lucena, Franklin Riet-Correa

**Affiliations:** 1 Graduate Program in Animal Science in the Tropics, School of Veterinary Medicine and Animal Science, Federal University of Bahia (UFBA), Salvador 40170-110, BA, Brazil; lais.caymmi@ufba.br (L.G.C.); anacarol.sns@gmail.com (A.C.S.N.S.); 2 School of Veterinary Medicine and Animal Science, Federal University of Bahia (UFBA), Salvador 40170-110, BA, Brazil; mucinhoferraro@hotmail.com (M.F.F.M.); paulavleal15@gmail.com (P.V.L.); tiagocpeixoto@yahoo.com.br (T.C.P.); 3 Veterinary Pathology Sector, Federal University of Recôncavo of Bahia (UFRB), Cruz das Almas 44380-000, BA, Brazil; lucianoanp@ufrb.edu.br (L.A.P.); jcoliveirafilho@gmail.com (J.C.d.O.-F.); 4 Veterinary Pathology Laboratory, Veterinary Hospital, Department of Veterinary Sciences, Center for Agricultural Sciences, Federal University of Paraíba (UFPB), Campus II, Areia 58397-000, PB, Brazil; lucena.rb@gmail.com

**Keywords:** brisket disease, chronic edema, cor pulmonale, bovines, pulmonary arterial hypertension, toxic plants

## Abstract

For nearly 30 years, a chronic disease causing severe chronic subcutaneous edema of unknown etiology has been affecting cattle in the Central–Northern and Central–Southern mesoregions of the State of Bahia, Brazil. In this research we investigated 15 outbreaks of the disease, from October 2023 to April 2025, to determine its etiopathogenesis, epidemiology, clinical signs, and pathology. The disease occurs during the dry season in areas of native forest. It is characterized by chronic subcutaneous edema, especially in the regions of the head, dewlap, chest, and thoracic limbs, and by hydropericardium, hydrothorax, ascites, and right ventricular dilation. Marked hypertrophy of smooth muscle cells in the tunica media of arteries and arterioles, sometimes with an eccentric, irregular, and asymmetric arrangement, were the main histologic lesions observed. The thicknesses of the media of pulmonary and heart arteries and arterioles of 10 affected cattle were significantly thicker than those from 10 control cattle. The tunica adventitia was thickened with increased deposition of collagen, and the intima was hyperplasic. The aorta and carotid arteries showed multifocal smooth muscle cell proliferation in the tunica media. It is concluded that the disease is due to right heart failure due to pulmonary arterial hypertension (chronic cor pulmonale). Epidemiological data and inspections of affected pastures suggest that the disease is caused by a toxic plant.

## Simple Summary

We report a disease characterized by severe subcutaneous edema in cattle in 15 farms of the State of Bahia, Brazil. The disease has affected bovines of different ages and of both sexes for at least 30 years in areas of native forests during periods of prolonged drought, when pastures have little forage availability. The first clinical signs were observed approximately two months after the introduction of cattle into the forest and the animals died after a clinical manifestation period of 5–15 days. Main clinical signs are marked progressive subcutaneous edema of the dewlap, thorax, forelimbs, and occasionally the head, together with pulsation and distention of the jugular vein, and progressive weight loss. At necropsies, the main lesions are hydrothorax, hydropericardium, ascites, cachexia, pallor of mucous membranes, and right ventricular dilatation. Histologically, the lungs have marked hypertrophy of smooth muscle cells in the medial layer of arteries and arterioles. The edema is due to right heart failure, secondary to pulmonary arterial hypertension caused by lesions of the blood vessels of the lung (chronic cor pulmonale). It is suggested that the disease is caused by a toxic plant.

## 1. Introduction

Cor pulmonale is a syndrome characterized by pathological alterations of the right ventricle due to pulmonary arterial hypertension [1,2]. The disease is characterized by hypertrophy and dilation of the right ventricle, secondary to a lung disease that causes pulmonary arterial hypertension, which also occurs in chronic lung diseases and several other conditions. The main clinical sign of the disease is generalized, severe, chronic, and progressive subcutaneous edema, which mainly affects the submandibular and abdominal regions, chest, and, occasionally, limbs. In the United States, pulmonary arterial hypertension represents significant economic losses for farmers in the mountainous regions of Colorado, Wyoming, Utah, and New Mexico [1]. The high incidence of this high-altitude illness suggests that the anatomical structure of cattle lungs predisposes them to the condition. Their lungs are relatively small and lobulated in comparison to their body weight, contributing to severe loss of functional lung capacity [1,2,3].

Sporadic cases of right-sided congestive heart failure, similar to brisket disease, have been reported also in feedlot cattle [4,5]. The disease occurs at a moderate altitude (800–1600 m) and the number of bovines treated for bovine respiratory diseases is associated with the occurrence of right-sided congestive heart failure [4,5,6]. The disease is also more frequent at higher elevations [5,6].

In Brazil, in the state of Mato Grosso do Sul, a clinical picture of dewlap edema has been reported in cattle [7]. The etiology remains undetermined, but the lesions suggest an IgE-mediated hypersensitivity process, similar to angioedema. The condition is seasonal, with high morbidity, and occurs during winter temperature drops. It primarily affects animals grazing *Urochloa* (*Brachiaria*) spp. pastures. Clinically, edema appears suddenly, without compromising the general condition of the affected animals. Histopathology reveals edema and eosinophilic infiltration in the subcutaneous tissue. The animals recover spontaneously [7].

Four groups of toxic plants are known to cause chronic edema in cattle in Brazil: (1) plants that cause chronic cardiac fibrosis, including *Ateleia glazioviana*, *Niedenzuella acutifolia*, and *Niedenzuella multiglandulosa*; (2) plants that cause hepatic fibrosis, including *Crotalaria* spp. and *Senecio* spp.; (3) plants that cause nephrosis, including *Combretum glaucocarpa*, *Amaranthus* spp., and *Metternichia princeps* [8]; and (4) *Crotalaria pallida*, the only plant described as a cause of pulmonary lesions with arterial hypertension [8,9].

**Figure 1:**
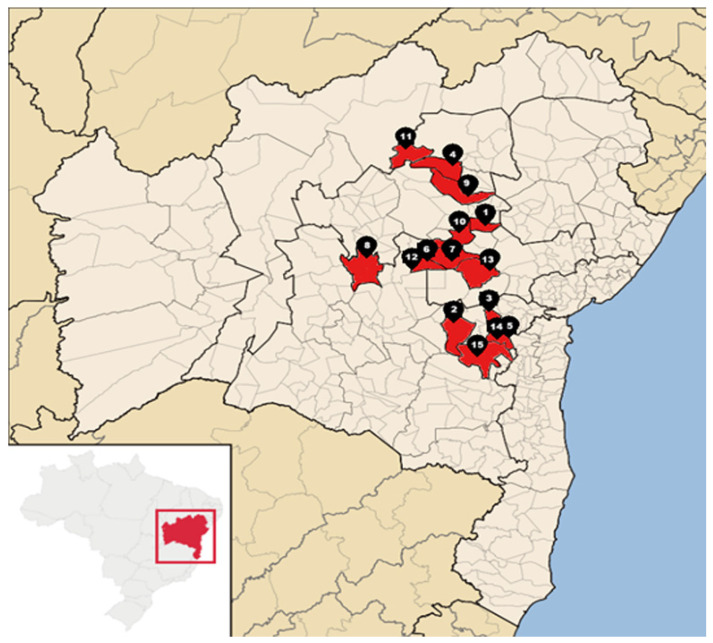
Map of the State of Bahia, Northeastern Brazil, highlighting in red the 13 municipalities where the 15 outbreaks of chronic edema disease in cattle occurred. The outbreaks studied occurred in the municipalities of Mairi (1), Maracás (2), Irajuba (3), Mirangaba (4), Jaguaquara (5), Lajedinho (6), Ruy Barbosa (7), and Seabra (8), while the reported outbreaks occurred in Jacobina (9), Mundo Novo (10), Umburanas (11), Lajedinho (12), Itaberaba (13), Jaguaquara (14), and Jequié (15).

For nearly 30 years, outbreaks of a disease of unknown etiology characterized by chronic subcutaneous edema, particularly affecting the head, dewlap, chest, and forelimbs, have been observed in cattle in an area of nearly 100.000 km^2^ in the Central–Northern and Central–Southern mesoregions of Bahia, affecting at least 12 municipalities [10] (Figure 1). This region has a semi-arid climate, with high temperatures and scarce and poorly distributed rainfall. The average annual temperature is around 24 °C (maximum 29.2 °C and minimum 20.2 °C). The average annual rainfall is 672 mm, ranging from 323 to 1147 mm, with a rainy period from November to April and a dry period from May to October [11]. The disease appears to be seasonal and occurs simultaneously on different farms and municipalities during prolonged dry periods, particularly between September and December. It manifests primarily in outbreaks, lasting one to three months, with a morbidity rate ranging from 5% to 11% and a fatality rate of up to 48%. Adult animals are the most affected, but severe cases of the disease also occur in weaned calves. Furthermore, there is no predisposition based on sex or breed, although it is more common in beef cattle [10]. The objective of this study was to describe the clinical, epidemiological, and pathological aspects of this disease, which is characterized by chronic edema associated with cor pulmonale in cattle in Bahia.

## 2. Material and Methods

Eight outbreaks of the disease were studied between October 2023 and April 2025. Additionally, another seven outbreaks reported by veterinarians and/or farmers were analyzed through observations of photographs, videos, and visits to farms.

Forty-six animals exhibiting edema were clinically examined, and blood and fecal samples were collected from these animals. Feces were collected from the rectal ampulla and subjected to egg count per gram of feces (EPG) using the MacMaster method. Blood samples were obtained by puncture of the coccygeal vein and collected in 5 mL tubes containing sodium ethylenediaminetetraacetate (EDTA) in 10% aqueous solution to perform hemograms (packed cell volume; number of red blood cells; hemoglobin concentration; mean packed cell volume; mean packed cell hemoglobin concentration; and number of leukocytes, rods, neutrophils, eosinophils, basophils, lymphocytes, and monocytes) [12]. Other blood samples were collected in 10 mL tubes containing clot activator to obtain serum after centrifugation at 1500 RCF for 10 min. The serum samples were transferred to Eppendorf’s^®^ microtubes and stored at −20 °C. Serum total protein, albumin, creatinine, and urea were determined by the colorimetric method, while the serum activities of aspartate aminotransferase (AST), gamma glutamyl transferase (GGT), and creatine kinase (CK) were obtained by the kinetic method using commercial kits from Bioclin^®^ (Belo Horizonte, Brazil) in an automatic biochemistry apparatus PKL 125 [13]. Globulin was calculated by the difference between total protein and albumin [13].

Seven autopsies were performed: three after natural death and four after euthanasia. Euthanasia was performed by intravenous administration of 2% xylazine hydrochloride (0.3 mg/kg) (Syntec^®^, Tamboré, Brazil), followed by 10% ketamine (5 mg/kg) (Syntec^®^) until lateral recumbency was achieved. Subsequently, 2% lidocaine (Bravet^®^, Engenho Novo, Brazil) was administered into the foramen magnum in a volume sufficient to induce cardiac arrest. At autopsies, fragments of brain, spinal cord, lung, heart, liver, kidney, spleen, esophagus, rumen, reticulum, omasum, abomasum, small intestine, large intestine, and subcutaneous tissue with edema were collected and fixed in 10% phosphate-buffered formalin. After fixation, these materials were embedded in paraffin, cut at a thickness of 5 µm, and stained with hematoxylin–eosin (HE). To evaluate muscle fiber hyperplasia in the arterioles, pulmonary samples from cattle affected by edema and from a healthy slaughterhouse bovine were submitted to immunohistochemical (IHC) analysis using an anti-actin (smooth muscle/a-SMA) antibody (M0851, 1:100 dilution, Dako, Carpinteria, CA, USA). Immunolabeling was visualized with 3,3′-diaminobenzidine (DAB), and sections were counterstained with Harris’ hematoxylin.

To determine the thickness of the medial layer of cardiac and pulmonary arteries and arterioles, five arteries or arterioles from the heart and five from the lung were randomly selected from 10 cattle with edema and 10 control cattle. The hearts and lungs of control animals were collected at a slaughterhouse located in the municipality of Feira de Santana from cattle of similar age (adults) and breed (Nelore) to those of the affected animals. Using a camera attached to a microscope, images of arteries and arterioles in the heart and lung were obtained using the Yais^®^ app and a 10× objective. These photographs were used for histomorphometry of the medial layer of the vessels. The images were analyzed using the ImageJ^®^ v1.54 application, in which the medial thickness was measured at four equidistant points (superior, inferior, right lateral, and left lateral). For statistical analysis, the data were transferred to an Excel^®^ spreadsheet and subjected to a normal distribution assessment. Nonparametric analysis was performed using the Mann–Whitney test for thickness of the media of the arteries and arterioles. The data were plotted on a scatterplot and analyzed using Graphprism^®^ software version 8.0.

The pastures where the affected animals were grazing were inspected. The suspicious plants observed in large amounts in different outbreaks were collected and sent to the Herbarium of the State University of Feira de Santana for botanical identification. In addition to the unknown suspicious plants, the toxic species already known were identified during the inspection of the pastures.

## 3. Results

Fourteen outbreaks occurred between October 2023 and December 2024 in the municipalities of Mairi, Maracás, Irajuba, Mirangaba, Jaguaquara, Ruy Barbosa, Jacobina, Umburanas, Lajedinho, Itaberaba, Jaguaquara, and Jequié during periods of prolonged drought, when pastures had very little forage availability and animals needed to be released into areas of native forest. One outbreak occurred in April 2025, in the municipality of Seabra, also during a dry season. The first clinical signs (subcutaneous edema) were observed approximately two months after the introduction of the animals into areas of native forest. Even so, the owner reported that the most severe presentation of the disease had also occurred on the farm at the end of the previous year. The periods of occurrence and other epidemiological characteristics observed in the 15 outbreaks are described in Table 1.

In outbreaks 7, 8, 9, and 12, the disease was observed in crossbred cattle, while in the other outbreaks, only Nelore animals were affected, but this was the only breed present on these farms. The age of the affected animals ranged from 2 to 4 years. However, in outbreak 6, five calves aged 4–6 months were observed with severe clinical signs, and of these, three rapidly evolved to death. The sex of the affected animals varied according to the type of production. However, on farms 3, 8, 9, and 12, where male and female cattle were raised on the same pasture, the disease was observed in both sexes.

Owners and employees of farms 1, 4, 6, 7, 8, 11, and 12 reported that the disease had occurred previously during prolonged periods of drought in cattle grazing on native forests. Some farmers also reported that the disease only appeared in animals brought from other regions and that animals born in the region were not affected.

**Figure 2:**
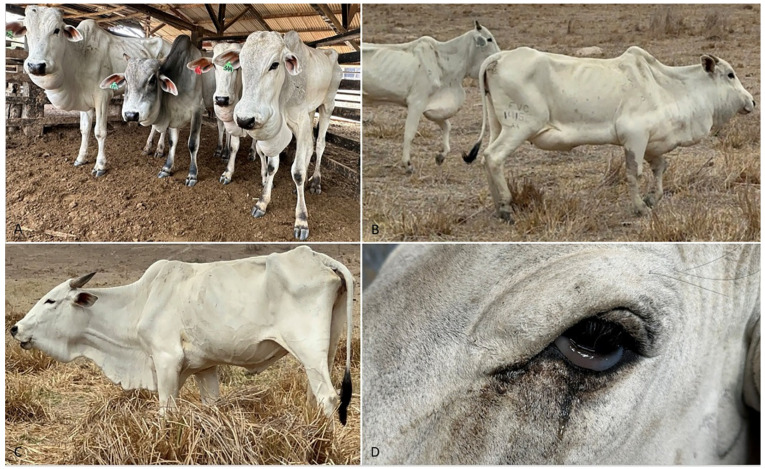
Bovines affected by chronic edema associated with cor pulmonale. (**A**) Subcutaneous edema of the submandibular region in four bovines. (**B**) Severe subcutaneous edema of the chest, scapula, and abdomen. (**C**) Edema of the face and submandibular region, severe weight loss, respiratory difficulties, and dilated jugular vein. (**D**) Edema of the conjunctiva.

The main clinical signs included marked progressive subcutaneous edema of the dewlap, thorax, forelimbs, and occasionally the head, together with marked distention and pulsation of the jugular vein, and progressive weight loss (Figure 2A–D). Edema of the inguinal region and udders and scleral edema (Figure 2D) were observed in some cases. Ventral edema was a common clinical sign in all animals examined; however, they usually presented with apathy, progressive weight loss, and bristly, coarse, dull hairs before the development of edema. Lethargy and anorexia were also observed from the onset of the clinical signs. Other signs included altered heart sounds and dyspnea.

Death occurred after a clinical manifestation period of 5–15 days; however, there were animals that showed gradual recovery when removed from the paddock with other pastures, and others that died suddenly even after the edema disappeared.

Results of hematologic values and serum biochemistry of 46 affected animals in farms 1–8 are presented in Table 2 and Table 3, respectively. Briefly, a normocytic normochromic anemia was the most frequent finding. The hematocrit, red blood cell count, and hemoglobin concentration were below the reference values in 50% (23/46) of the cattle examined. In the serum biochemistry, hypoproteinemia was observed in 60% (28/46) of the animals evaluated, hypoalbuminemia in 86% (40/46), and hyperglobulinemia in 84% (39/46). In the evaluation of renal function, mean urea levels were below the reference value in 26% (12/46) of cases. On the other hand, creatinine levels were elevated in 28% (13/46) of affected cattle, but both parameters were not related. The serum liver enzyme activities showed decreased serum AST activity in 28% (13/46) of the animals and elevation in 22% (10/46) of the cases. Serum GGT activities were elevated in 72% (33/46) of the cattle examined, representing the most frequent alteration. Serum CK activities were elevated in 26% (12/46) of the animals examined. In the stool examination, there was the absence of or less than 50 helminth eggs per gram of feces.

**Figure 3:**
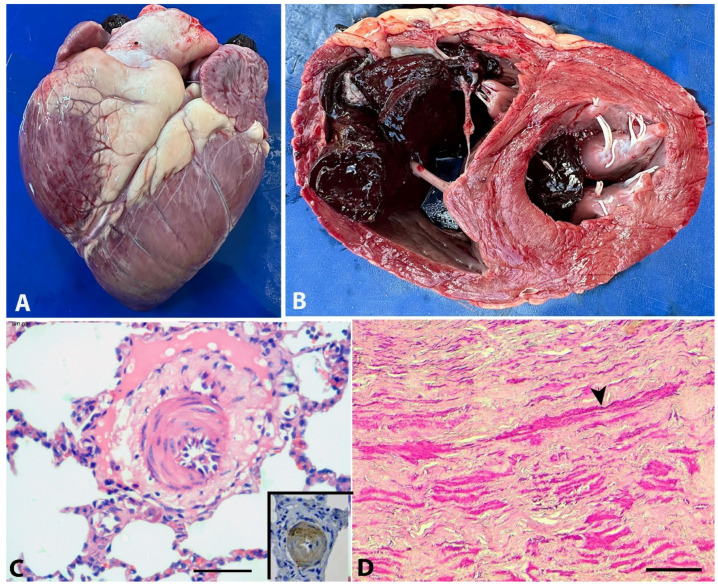
Bovines affected by chronic edema associated with cor pulmonale. (**A**) Heart showing enlarged and rounded right ventricle. (**B**) Transversal cut of the heart showing enlarged right ventricle and clotted blood in the left ventricle suggesting uncomplete rigor due to myocardial degeneration. (**C**) Arteriole in lung with tunica media irregularly thickened by hypertrophy of myocytes. Tunica adventitia has increased deposition of collagen and edema. The intima is hyperplastic H-E. Bar = 50 µm. Inset: Positive immunohistochemistry for smooth muscle cells (α-SMA), showing intense labeling in the hyperplastic arteriolar wall. Counterstained with Harris hematoxylin. (**D**) Aorta of an affected cattle showing multifocal proliferation of smooth muscle cells (arrow). H-E, bar = 100 µm.

Postmortem examinations were performed on seven animals. Gross lesions, present in all animals, included subcutaneous edema, particularly in the thorax, dewlap, and facial regions; and hydrothorax, hydropericardium, ascites, cachexia, and pallor of the mucous membranes and organs. Hydrothorax was the most pronounced effusion encountered. Edema of the abomasum and small intestine, mainly in the duodenum, was also observed. Pulmonary lesions consisted of pulmonary edema with interlobular septal thickening. Cardiac findings included right ventricular dilatation (Figure 3A,B). Serous fat atrophy was observed in some animals. The livers were moderately enlarged, with rounded edges and dark red or yellowish coloration. An accentuated lobular pattern (nutmeg appearance) was observed in four animals. The kidneys appeared normal, except for some pallor or redness.

**Figure 4:**
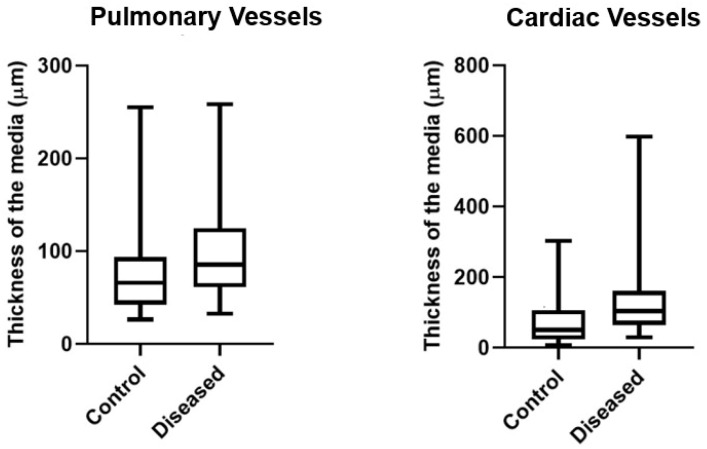
Boxplots showing the thickness of pulmonary (**left**) and cardiac (**right**) arterioles and arteries of affected and unaffected cattle. In both organs, the thicknesses of the media of affected animals are significantly greater (*p* < 0.05) than those of unaffected control animals.

Histologically, in the lungs, there was marked hypertrophy of smooth muscle cells in the medial layer of arteries and arterioles, sometimes with an eccentric, irregular, and asymmetric arrangement (Figure 3C). On immunohistochemistry, the hyperplastic cells in the arteriolar media were intensely positive for α-SMA (Figure 3, Inset). The tunica adventitia was thickened with increased deposition of collagen, and the intima was hyperplasic with endothelial cell proliferation (Figure 3C). Additionally, degeneration of endothelial cells, duplication of the elastic lamina, periarteriolar fibrosis, fibrinoid necrosis, or perivascular edema were occasionally observed. The alveolar septa were moderately thickened, occasionally with connective tissue proliferation and edema; multifocal hyperplasia of type II pneumocytes was also observed. Similar lesions were observed in some arterioles and arteries of the heart. The aorta and carotid arteries showed multifocal smooth muscle cell proliferation in the tunica media (Figure 3D). Both the lung and heart arteries and arterioles of the affected cattle were significantly thicker (*p* < 0.05) than those of the control animals (Figure 4). Hepatic findings included vacuolization of hepatocytes, centrilobular or diffuse congestion, and centrilobular necrosis and loss of hepatocytes with replacement by fibrous tissue. In two animals, few arteries had marked hypertrophy of smooth muscle cells in the tunica media.

Renal lesions involved glomerular changes characterized by proliferation of mesangial cells, occasionally with cytoplasmic vacuoles in the mesangial cells and moderate vascular congestion. In some glomeruli, cell proliferation resulted in synechiae with Bowman’s capsule, and the affected glomeruli occasionally showed fibroplasia (glomerulosclerosis). Multifocal areas of vacuolar degeneration and tubular epithelial necrosis were observed in two animals. Dilated tubules with epithelial regeneration were also observed occasionally. In one animal, few arteries had marked hypertrophy of smooth muscle cells in the tunica media.

## 4. Discussion

The clinical–pathological picture reported in this study is characterized by generalized subcutaneous edema and right ventricular dilation with histological lesions of pulmonary arteries and arterioles, in particular hypertrophy of the smooth muscle cells of the medial layer. These lesions suggest that the edema is due to right heart failure, secondary to pulmonary arterial hypertension caused by lesions of the blood vessels of the lung (chronic cor pulmonale). These results rule out the possibility of the disease being caused by any of the plants that cause edema as a result of liver fibrosis, kidney failure, or cardiac fibrosis mentioned in the introduction [8].

In clinical pathology, although several altered values were recorded, none of these alterations were consistent in all animals tested, which suggests that they were not the primary cause of the disease. They were probably a consequence of anorexia and nutritional deficiencies, since the disease occurs in the dry season, when there is a shortage of forage. In some cases, it may have been due to hepatic lesions, which were observed in some animals, probably as a consequence of heart failure.

We suggest that the chronic bovine edema disease (cor pulmonale) reported here is caused by constriction of the blood vessels in the lungs with consequent increase in pulmonary arterial pressure and secondary lesions of the right ventricle. It is clinically and pathologically very similar to that known as Brisket disease, which occurs in areas located at high altitudes (around 2000 m) [3], although the disease has been reported at lower altitudes (1369 m) in western Nebraska [2]. However, the municipalities in Bahia where the disease has been observed are located at much lower altitudes (ranging from 368 m to 1.069 m), making it unlikely that the etiology is related to altitude. Plants of the *Astragalus* and *Oxytropis* genera (locoweeds), which contain swainsonine, may exacerbate the hypoxic condition in animals susceptible to pulmonary hypertension [14]. In Northeast Brazil, plants of the *Ipomoea* genus, which also contain swainsonine, are well known, but no reports of edema caused by these plants exist; they are typically associated with neurological signs [8,15]. Furthermore, no plants of this genus were found on the farms where the outbreaks occurred.

Lesions of arteries and arterioles were also observed in other organs, mainly in the aorta and carotid arteries that showed multifocal smooth muscle cell proliferation. The clinical significance of these arterial lesions, observed in of all necropsied animals, is unknown. In humans, the proliferation and degradation of smooth muscle cells promote aortic diseases including aortic aneurysms and aortic dissection [16].

The epidemiological characteristics of the disease (occurrence in specific areas of native forest; during the dry season; and affecting cattle of different ages, sexes, and breeds) suggest that the disease is caused by a toxic plant. In an experiment, *Fredericia cinerea* found on farms where the disease occurs was administered in daily doses of 40 g/kg to a bovine for 60 days and no clinical signs were observed [10]. Another plant, *Banisteriopsis oxyclada*, was administered to one bovine at a dose of 20 g/kg for 40 days. The animal presented weight loss, loose feces (dirty perineum), jugular engorgement, and positive venous pulse, but no edema was observed [17]. New experiments should be carried out with other plants found in areas where the disease occurs.

Certain species of *Crotalaria* that contain pneumotoxic pyrrolizidine alkaloids that induce pulmonary hypertension and associated vascular changes are often used as a model for experimental pulmonary hypertension in laboratory animals [18]. In Brazil, lung lesions, such as thickening of the alveolar walls and arterioles with reduction in the lumen and periarteriolar fibrosis, were observed in animals experimentally poisoned with *Crotalaria mucronata* [9]. However, no significant amounts of *Crotalaria* species were found in the areas where the disease occurred.

Another disease that induces pulmonary arterial hypertension is poisoning by *Pimelea* spp., which contains a toxin (simplexin) that causes contraction of the muscular walls of blood vessels in the lungs. The disease occurs in Australia, where it is known as St. George disease. Vasoconstriction triggers an increase in pressure followed by effusion, right heart failure, and subcutaneous edema. In the liver, the toxin induces expansion of the sinusoids and portal venules (peliosis hepatis) and also affects the intestinal tract, causing diarrhea [19]. *Pimelea* spp. do not occur in Brazil, and the clinical–pathological picture induced by simplexin, despite including right heart failure, appears to be different from that observed in the cattle in this study.

Parasitic causes of edema due to hypoproteinemia, including hemonchosis, ostertagiasis, and fasciolosis, were ruled out by the results of fecal analyses and necropsies. Fasciolosis has not been reported in the region. Heartwater, caused by *Cowdria ruminantium*, does not occur in Brazil, nor does its vector (*Amblyomma variegatum*). Additionally, the neurological signs characteristic of heartwater were not observed, nor was the presence of the agent in blood vessels during histological examination [20].

In conclusion, the disease results from pulmonary arterial hypertension induced by lesions of the pulmonary arterioles, with subsequent right ventricular failure. Such arterial lesions are caused, probably, by an unknown toxic plant. The disease causes significant economic losses in a large region of the state of Bahia, and it is necessary to know its etiology in order to determine prophylaxis and control measures. Farm visits during disease outbreaks to identify plants suspected of causing the disease and the administration of these plants to experimental cattle are necessary to determine the cause of the progressive generalized edema reported in this article.

## Figures and Tables

**Table 1:** Epidemiological data on outbreaks of chronic edema disease in cattle in the State of Bahia, Northeastern Brazil.

Outbreak	Month/Year	Municipality	N° of Cattle	N° Affected Cattle	N° Dead Cattle	Age of Affected Cattle	Sex of Affected Cattle
1	Oct/2023	Mairi	415	11 (2, 6%)	5 (45%)	3 years	M
2	Nov/2023	Maracás	160	22 (13, 7%)	9 (41%)	4 years	F
3	Nov/2023	Irajuba	390	10 (2, 5%)	7 (70%)	3–4 years	M and F
4	Dec/2023	Mirangaba	60	10 (16, 7%)	4 (40%)	3 years	F
5	Dec/2023	Jaguaquara	450	20 (4, 4%)	6 (30%)	3 years	F
6	Dec/2023	Lajedinho	2.000	50 (2, 5%)	15 (30%)	3–4 years 4–6 m	F
7	Dec/2024	Ruy Barbosa	300	10 (3, 3%)	4 (40%)	2–3 years	M
8	Apr/2025	Seabra	11	4 (36, 4%)	1 (25%)	2–3 years	M and F
9	Dec/2023	Jacobina	200	3 (1, 5%)	3 (100%)	2–4 years	M
10	Dec/2023	Mundo novo	93	7 (7, 5%)	4 (57%)	2–3 years	M and F
11	Dec/2023	Umburanas	32	4 (12%)	3 (75%)	2 years	M
12	Dec/2023	Lajedinho	67	3 (4%)	1 (33%)	2–3 years	F
13	Dec/2023	Itaberaba	123	10 (8, 1%)	7 (70%)	2–3 years	M and F
14	Dec/2023	Jaguaquara	45	4 (9%)	2 (50%)	2–3 years	F
15	Dec/2023	Jequié	80	8 (10%)	5 (62%)	2–3 years	M

**Table 2:** Mean, standard deviations, and reference values of hematological parameters evaluated in cattle with chronic edema in Bahia.

Outbreaks	1	2	3	4	5	6	7	8	Reference Values *
PCV (%)	23 ± 3.4	25 ± 5.1	32 ± 3	29 ± 5.7	34 ± 5.5	21 ± 3.9	22 ± 3.2	24 ± 1.7	24–46
RBC (10^6^/μL)	4.9 ± 1	5.5 ± 1.5	6.8 ± 1.5	6.6 ± 1.4	6.5 ± 1.1	4.2 ± 0.9	5.0 ± 0.9	3.5 ± 0.3	5–10
Hb (g/dL)	8 ± 1.1	8 ± 1.7	11 ± 1	9.5 ± 1.9	11 ± 1.8	7 ± 1.3	7.5 ± 1.0	7.9 ± 0.5	8–15
MPCV (fL)	49 ± 8.8	47 ± 5.9	49 ± 8	44 ± 3	50 ± 2.8	52 ± 9.2	45 ± 4.7	67 ± 2.8	40–60
MPVH (%)	33 ± 0	33.1 ± 0.1	33 ± 0	33 ± 0	33 ± 0	33 ± 0	33 ± 0	33.1 ± 0	30–36
Leukocytes (μL)	6080 ± 360	7950 ± 3480	9700 ± 2700	5250 ± 1740	8240 ± 3450	6235 ± 2581	6730 ± 1628	4970 ± 1400	4000–12,000
Rods (μL)	109 ± 326	10 ± 32	26 ± 45	49 ± 88	0 ± 0	104 ± 115	40 ± 64	305 ± 68	0–120
Neutrophils (μL)	1410 ± 595	2640 ± 1710	3970 ± 3900	1410 ± 1080	2300 ± 429	1270 ± 1040	2950 ± 1190	2160 ± 241	600–4000
Eosinophils (μL)	310 ± 240	360 ± 300	110 ± 130	70 ± 90	460 ± 470	385 ± 360	220 ± 235	37 ± 33	600–2400
Basophils (μL)	43 ± 60	0 ± 0	0 ± 0	0 ± 0	0 ± 0	0 ± 0	0 ± 0	0 ± 0	0–200
Lymphocytes (μL)	3460 ± 510	4630 ± 2250	5140 ± 2900	2670 ± 1040	4340 ± 1390	4170 ± 1720	2670 ± 670	2260 ± 151	2500–7500
Monocytes (μL)	455 ± 290	330 ± 225	330 ± 260	840 ± 1060	210 ± 110	500 ± 400	860 ± 420	207 ± 151	25–840

PCV: Packed cell volume; RBC: red blood cell; Hb: hemoglobin; MPCV: mean packed cell volume; MPCH: mean packed cell hemoglobin concentration. * Feldman et al. [12].

**Table 3:** Mean, standard deviations, and reference values of serum biochemical parameters evaluated in cattle with chronic edema in Bahia.

Outbreaks	1	2	3	4	5	6	7	8	Reference Values *
TP (g/dL)	6.4 ± 0.5	6.6 ± 0.6	8 ± 0.3	6.9 ± 1.3	7 ± 0.4	6.6 ± 0.7	6.7 ± 0.3	5.6 ± 0.6	6.7–7.4
Albumin (g/dL)	2.5 ± 0.2	2.4 ± 0.2	2.9 ± 0.2	2.4 ± 0.4	2.8 ± 0.4	2.4 ± 0.4	2.4 ± 0.1	3.3 ± 0.5	3–3.6
Globulin (g/dL)	3.9 ± 0.3	4.1 ± 0.5	5.0 ± 0.2	4.5 ± 1.6	4.2 ± 0.4	4.1 ± 0.5	4.3 ± 0.3	2.5 ± 0.2	3–3.5
Urea (mg/dL)	31 ± 12	34 ± 10	29 ± 6	25 ± 3	21 ± 3	40 ± 12	30 ± 9	20 ± 6.4	23–58
Creatinine (mg/dL)	2.3 ± 0.6	1.6 ± 0.3	1.8 ± 0.4	1.4 ± 0.2	1.8 ± 0.3	2.1 ± 0.3	1.5 ± 0.3	1.2 ± 0.1	1–2
AST (UI)	120 ± 38	98.5 ± 13	78 ± 7.9	77 ± 29.5	97.4 ± 45.9	113 ± 47.5	52 ± 9.8	186 ± 31.6	78–132
GGT (μL)	17 ± 1.9	17.8 ± 2.7	30 ± 8.1	17 ± 2.3	22 ± 7	19 ± 5.2	30 ± 9.5	17.5 ± 0.5	6.1–17.4
CK (μL)	272 ± 180	211 ± 242	79 ± 18	133 ± 20	238 ± 250	190 ± 170	337 ± 330	1434 ± 1109	35–280

TP: Total serum protein; AST: aspartate aminotransferase; GGT: gamma-glutamyl transferase; CK: creatine kinase. * Reference values: Kaneko et al. [13].

## Data Availability

Generative AI was not used in the writing of this manuscript.
